# Ethylene Inhibitors Enhance Shoot Organogenesis of Gloxinia (*Sinningia speciosa*)

**DOI:** 10.1100/2012/859381

**Published:** 2012-10-17

**Authors:** Soo Cheon Chae, Haeng Hoon Kim, Sang Un Park

**Affiliations:** ^1^Department of Horticultural Science, College of Industrial Sciences, Kongju National University, 1 Daehoe-ri, Yesan-kun, Chungnam 340-720, Republic of Korea; ^2^Department of Well-Being Resources, Sunchon National University, 413 Jungangno, Suncheon, Jeollanam-do 540-742, Republic of Korea; ^3^Department of Crop Science, College of Agriculture and Life Sciences, Chungnam National University, 99 Daehangno, Yuseong-gu, Daejeon 305-764, Republic of Korea

## Abstract

Shoot organogenesis and plant regeneration in *Sinningia speciosa* were improved using ethylene inhibitors. The leaf explants were cultured on initial shoot regeneration media (MS media with BAP at 2 mg/L + NAA at 0.1 mg/L) supplemented with different concentrations of aminoethoxyvinylglycine (AVG), cobalt chloride (CoCl_2_), and silver thiosulphate (STS). The addition of AVG, CoCl_2_, and STS significantly improved the regeneration frequency giving higher shoots per explant and longer shoot length. The highest shoot growth was found when STS at 5 mg/L was incorporated with generation medium, performing highest regeneration frequency with highest number of shoots. This treatment (STS at 5 mg/L) produced 40% more shoots per explant compared to control followed by STS at 10 mg/L with increasing 37% more shoots compared to control. In the cases of AVG and CoCl_2_ the highest shoot number per explant was found at 1 mg/L. Treated with AVG and CoCl_2_ at 1 mg/L increased shoot number by 16 and 12%, respectively, compared to control. Ethylene inhibitors could be used as a possible micropropagation and plant transformation protocol in *S. speciosa* for plant regenerations.

## 1. Introduction

*Sinningia speciosa* Baill, commonly known in the horticultural trade as gloxinia, is a tuberous member of the flowering plant family Gesneriaceae. The common name has persisted since its original introduction to cultivation from Brazil in 1817 as *Gloxinia speciosa*. The name florist's gloxinia is sometimes used to distinguish it from the rhizomatous species now included in the genus *Gloxinia*. This species produces single or double flowers with a variety of colors. Generally its propagation can be done by leaf, stem, rhizome, seed, and crown cuttings from a mature plant after blooming. It takes approximately 6 to 7 months for commercial production of a blooming gloxinia [1, 2].

Successful shoot organogenesis technique for plant regeneration depends on the proper establishment of medium components, a suitable explant, and control of the physical environment [3, 4]. One of the most important factors of physical environment in plant tissue culture is ethylene (C_2_H_4_), a gaseous plant hormone that plays an important role in plant growth and development [5]. Same research was done in the past using ethylene inhibitors, that is, aminoethoxyvinylglycine (AVG), cobalt chloride (CoCl_2_), benzyl isothiocyanate (BITC), aminocarboxypropionic acid, 1-methylcyclopropene (1-MCP), polyamines, silver nitrate (AgNO_3_), 3,4,5-trichlorophenol, salicylic acid (2-hydroxybenzoic acid), and silver thiosulphate (STS), for promoting shoot organogenesis in several plant species which has been reviewed by Kumar et al. [6]. 

Recently some studies especially for *in vitro* plant regeneration were done in gloxinia using leaf explant culture [7–10] and even direct regeneration of floral buds from sepal segments has been reported [11, 12]. In this paper, we report the establishment of an improved method for plant regeneration from the leaf explants of *S. speciosa. *We examined for the first time the influence of ethylene inhibitors (STS, AVG, and CoCl_2_) on shoot organogenesis of *S. speciosa*. 

## 2. Materials and Methods

### 2.1. Plant Material

Seeds of* Sinningia speciosa *were surface-sterilized with 70% (v/v) ethanol for 1 min and 2% (v/v) sodium hypochlorite solution for 10 min, then rinsed three times in sterilized water. Five seeds were placed on 25 mL of agar-solidified culture medium in Petri dishes (100 × 15 mm). The basal medium consisted of salts and vitamins of MS [13] medium and solidified with 0.7% (w/v) agar. The medium was adjusted to pH 5.8 before adding agar and then sterilized by autoclaving at 121°C for 20 min. The seeds were germinated in a growth chamber at 25 ± 1°C under standard cool white fluorescent tubes with a flux rate of 35 *μ*mol s^−1 ^m^−2^ and a 16-h photoperiod.

### 2.2. Shoot Organogenesis

Young leaves of* Sinningia speciosa *were taken from *in vitro* grown plants. Leaves were cut aseptically at the ends, into sections of approximately 7 × 7 mm^2^ in size. Explants were placed on the MS medium and solidified with 0.3% (w/v) Gelrite. Seven explants were cultured in each Petri dish. The pH of medium was adjusted to 5.8 before adding Gelrite. The media were sterilised by autoclaving at 1.1 kg cm^−2^ (121°C) for 20 min. Previously, we established gloxinia shoot induction medium consisting of MS salts and vitamins, 30 g/L sucrose, 3 g/L Gelrite, 2 mg/L 6-benzylaminopurine (BAP), and 0.1 mg/L NAA (1-naphthalene-acetic acid) [10]. For improvement of shoot regeneration of gloxinia, the shoot induction medium was optimized by testing the effect of different concentrations of ethylene inhibitors (0, 1, 5, 10, and 20 mg/L aminoethoxyvinylglycine, cobalt chloride, and silver thiosulphate). Cultures were maintained at 25 ± 1°C in a growth chamber with a 16-h photoperiod under standard cool white fluorescent tubes (35 *μ*mol s^−1 ^m^−2^) for 6 weeks. 

### 2.3. Rooting and Acclimatization of Regeneration Plants

Regenerated shoots (around 1 cm long) were placed in MS medium. The medium was solidified with 3 g/L Gelrite and dispensed at 30 mL per Magenta box and four shoots were cultured in each box. Regenerated shoots were incubated at 25 ± 1°C in a growth chamber with a 16-h photoperiod under standard cool white fluorescent tubes (35 *μ*mol s^−1 ^m^−2^) for 5 weeks. After five weeks, the rooted plants were washed with tap water to remove Gelrite, transferred to pots containing autoclaved vermiculite, and covered with polyethylene bags for one week to maintain high humidity. The plants were then transferred to soil and maintained in a growth chamber with a 16-h photoperiod, and a night/day temperature of 18/20°C for 2 weeks. These hardened plants were then transferred to the greenhouse.

## 3. Results 

Recently an improved and effective method for the *in vitro *plant regeneration of *S. speciosa *was reported by Park et al. [10]. We established optimal medium for plant regeneration of *S. speciosa* including BAP (2 mg/L) and NAA (0.1 mg/L) resulting in the highest efficiency in shoot regeneration per explant and in the greatest shoot growth. For investigating the influence of ethylene inhibitors on shoot regeneration of *S. speciosa*, the leaf explants were cultured on initial shoot regeneration media (MS media supplemented with BAP at 2 mg/L + NAA at 0.1 mg/L) that included 0, 1, 5, 10, and 20 mg/L aminoethoxyvinylglycine (AVG), cobalt chloride (CoCl_2_), and silver thiosulphate (STS).

The addition of AVG, CoCl_2_, and STS significantly improved the regeneration frequency giving higher shoots per explants and longer shoot length (Table 1). Shoot growth increased with increasing concentrations of STS up to 5 mg/L, but thereafter decreased as the concentrations increased. In this study, the highest shoot growth was found when the generation medium (MS media with BAP at 2 mg/L + NAA at 0.1 mg/L) was supplemented with STS at 5 mg/L, performing 91% regeneration frequency with highest number of shoots (17.2) in each explant (Table 1). This treatment (STS at 5 mg/L) produced 40% more shoot per explant compared to control. STS at 10 mg/L produced the second highest shoot number (16.8) that represents 37% more shoots compared to control (Table 1). The shoot number from each explant was 25 and 20% higher at STS at 5 mg/L compared to the treatment of AVG and CoCl_2_, respectively, which had the highest shoot number at 1 mg/L of both AVG and CoCl_2_ (Table 1). In the cases of AVG and CoCl_2_ the highest shoot number per explant was found at 1 mg/L. Treated with AVG and CoCl_2_ at 1 mg/L increased shoot number by 16 and 12%, respectively, compared to control (Table 1). Further increasing the concentration of AVG and CoCl_2_, the number of shoots per explant was reduced. The tallest plant was found in the treatment of AVG at 10 mg/L, which represents 49% increase in height compared to control. To induce root formation, the regenerated shoots were transferred to MS without growth hormones. The rooting started to initiate after 3 weeks from regenerated shoots, and more than 90% of the shoots contained roots after 5 weeks. The regenerated plants which contained root were washed in tap water to remove Gelrite and transferred into pots. The plants were allowed to grow in a growth chamber at 25 ± 1°C with a 16-h photoperiod for two weeks. Pots were covered with a plastic bag to maintain high humidity conditions for two weeks. The survival rate of regenerated plants was 80% and flowered within 3 months.

## 4. Discussion 

The shoot growth increased when the STS concentrations changed from 1 mg/L to 5 mg/L, but thereafter decreased with increasing STS concentrations. In this study, the highest regeneration frequency (91%) and number of shoots per explant (17.2) were observed when the regeneration medium was supplemented with STS at 5 mg/L (Table 1). This treatment produced 40% more shoots per explant compared to the control. The shoot number per explant was 25% and 20% higher at an STS supplementation of 5 mg/L when compared to the shoot number in the presence of AVG and CoCl_2_, respectively, at 1 mg/L. Further increases in the concentration of AVG and CoCl_2_ reduced the number of shoots per explant. Ethylene is produced during cell division *in vitro* and acts as a growth inhibitor. Further, the use of the ethylene inhibitors STS or AVG has been shown to increase the frequency of successful plant regeneration in apricot cultivars [14]. Moreover, the addition of AgNO_3_ and AVG to the medium was reported to markedly enhance regeneration frequency and the number of shoots per explant in *Punica granatum* L. [15]. The promotive effect of AgNO_3_, and AVG on *in vitro* shoot regeneration from cotyledons of *Brassica campestris* spp. *pekinensis* has also been reported [16]. During cell division *in vitro* ethylene is produced and it is very well known that ethylene acts as a growth inhibitor. It was reported that AgNO_3_ (ethylene inhibitor) inhibits the binding of ethylene during cell division [17]. Kumar et al. [6] reviewed the use of silver nitrate in plant regeneration and concluded that this chemical promoted growth of plants. Other species, including cucumber [17], *Brassica* [18], and coffee [19] have also been found to be affected by silver nitrate.

It is believed that plant regeneration protocols are an essential part of plant genetic transformation and lead to plant improvement. Currently, shoot organogenesis is used in *in vitro* plant regeneration as a most widely used method in transformation systems. This regeneration protocol has succeeded for *Sinningia speciosa. *The ethylene inhibitors AVG, CoCl_2_, and STS significantly promoted the shoot regeneration frequency of gloxinia. These results will allow the genetic improvement of *Sinningia speciosa *and other flower species. 

## Figures and Tables

**Table 1:** Effect of different concentrations of ethylene inhibitors on on shoot regeneration and growth from leaf cultures of *Sinningia speciosa* after 6 weeks in culture on regeneration medium (MS medium with 2.0 mg/L BA and 0.1 mg/L NAA).

Ethylene inhibitors* (mg L^−1^)		Regeneration frequency** (%)	Number of shoots per explant**	Shoot length^a^ (cm)
Control	0	72	12.3 ± 0.8	1.21 ± 0.1

AVG	1	85	14.3 ± 1.2	1.23 ± 0.2
	5	75	13.5 ± 0.9	1.6 ± 0.2
	10	64	10.6 ± 0.7	1.8 ± 0.2
	20	58	8.2 ± 0.4	1.1 ± 0.1

CoCl_2_	1	79	13.8 ± 0.7	1.36 ± 0.1
	5	72	11.8 ± 0.9	1.18 ± 0.1
	10	62	9.2 ± 0.6	0.85 ± 0.1
	20	45	5.9 ± 0.4	0.57 ± 0.1

STS	1	77	13.1 ± 0.9	1.18 ± 0.1
	5	91	17.2 ± 1.6	1.27 ± 0.2
	10	89	16.8 ± 1.2	1.25 ± 0.2
	20	63	11.4 ± 0.8	0.84 ± 0.1

^∗^Basal medium consisted of MS salts and vitamins, 30 g L^−1^ sucrose, and 2 mg/L BAP, solidified with 7 g/L Phytagar.

^∗∗^From 100 leaf explants tested.

^a^Values represent the mean ± standard deviation of 50 shoots.

## References

[1] Chautems A, Baracho GS, Siqueira Filho JA (2000). A new species of Sinningia (Gesneriaceae) from northeastern Brazil. *Brittonia*.

[2] Zaitlin D, Pierce A (2010). Nuclear DNA content in Sinningia (Gesneriaceae); Intraspecific genome size variation and genome characterization in *S. speciosa*. *Genome*.

[3] Brown DC, Thorpe TA, Vasil IK (1986). Plant regeneration by organogenesis. *Cell Culture and Somatic Cell Genetics of Plants*.

[4] Thorpe TA, Bhojwani SS (1990). The current status of plant tissue culture. *Plant Tissue Culture: Applications and Limitations*.

[5] Yang SF, Hoffman NE (1984). Ethylene biosynthesis and its regulation in higher plants. *Annual Review of Plant Physiology*.

[6] Kumar PP, Lakshmanan P, Thorpe TA (1998). Regulation of morphogenesis in plant tissue culture by ethylene. *In Vitro Cellular and Developmental Biology*.

[7] Scaramuzzi F, Apollonio G, D’Emerico S (1999). Adventitious shoot regeneration from *Sinningia speciosa* leaf discs in vitro and stability of ploidy level in subcultures. *In Vitro Cellular and Developmental Biology*.

[8] Nhut AT, Nguyet NA, Phuc HT (2006). Primary designs of tube-shaped nylon film culture system in shoot regeneration of Sinningia spp. Leaf explants. *Proceedings of International Workshop on Biotechnology in Agriculture*.

[9] Xu QL, Hu Z, Li CY, Wang XY, Wang CY (2009). Tissue culture of *Sinningia speciosa* and analysis of the in vitro-generated tricussate whorled phyllotaxis (twp) variant. *In Vitro Cellular and Developmental Biology*.

[10] Park EH, Bae H, Park WT, Kim YB, Chae SC, Park SU (2012). Improved shoot organogenesis of gloxinia (*Sinningia speciosa*) using silver nitrate and putrescine treatment. *Plant Omics Journal*.

[11] Pang JL, Wang LL, Hu JQ, Liang HM (2004). Effect of gibberellin on direct regeneration of floral buds from young flower buds in *Sinningia speciosa* Hiern. *Acta Biologiae Experimentals Sinican*.

[12] Pang JL, Wang LL, Hu JQ, Xiang TH, Liang HM (2006). Synergistic promotion of gibberellin and cytokinin on direct regeneration of floral buds from in vitro cultures of sepal segments in *Sinningia speciosa* Hiern. *In Vitro Cellular and Developmental Biology*.

[13] Murashige T, Skoog F (1962). A Revised medium for rapid growth and bio assays with tobacco tissue cultures. *Physiologia Plantarum*.

[14] Burgos L, Alburquerque N (2003). Ethylene inhibitors and low kanamycin concentrations improve adventitious regeneration from apricot leaves. *Plant Cell Reports*.

[15] Naik SK, Chand PK (2003). Silver nitrate and aminoethoxyvinylglycine promote in vitro adventitious shoot regeneration of pomegranate (*Punica granatum* L.). *Journal of Plant Physiology*.

[16] Chi GL, Pua EC, Goh CJ (1991). Role of ethylene on de novo shoot regeneration from cotyledonary explants of *Brassica campestris* ssp. pekinensis (Lour) olsson in vitro. *Plant Physiology*.

[17] Mohiuddin AKM, Chowdhury MKU, Abdullah ZC, Napis S (1997). Influence of silver nitrate (ethylene inhibitor) on cucumber in vitro shoot regeneration. *Plant Cell, Tissue and Organ Culture*.

[18] Akasaka-Kennedy Y, Yoshida H, Takahata Y (2005). Efficient plant regeneration from leaves of rapeseed (Brassica napus L.): The influence of AgNO_3_ and genotype. *Plant Cell Reports*.

[19] Kumar V, Ramakrishna A, Ravishankar GA (2007). Influence of different ethylene inhibitors on somatic embryogenesis and secondary embryogenesis from Coffea canephora P ex Fr. *In Vitro Cellular and Developmental Biology*.

